# COVID-19-Pandemie: Präferenzen und Barrieren für die Disseminierung von Evidenzsynthesen: Befragung des intensivmedizinischen Personals in Deutschland

**DOI:** 10.1007/s00101-021-01037-z

**Published:** 2021-09-21

**Authors:** Christian Seeber, Maria Popp, Joerg J. Meerpohl, Falk Fichtner, Anne Werner, Christoph Schmaderer, Christopher Holzmann-Littig, Steffen Dickel, Clemens Grimm, Onnen Moerer, Peter Kranke, AP6 CEOsys, Anke Steckelberg, Astrid Viciano, Carina Benstöm, Georg Holger Wormer, Jörg Wipplinger, Julia Krieger, Karolina Dahms, Kelly Ansems, Julia Lühnen, Marcus Anhäuser, Patrick Meybohm, Stephanie Weibel, Marjo Wijnen-Meijer

**Affiliations:** 1 Klinik und Poliklinik für Anästhesiologie und Intensivtherapie, Universitätsklinikum Leipzig AöR, Liebigstraße 20, 04103 Leipzig, Deutschland; 2 Klinik und Poliklinik für Anästhesiologie, Intensivmedizin, Notfallmedizin und Schmerztherapie, Universitätsklinikum Würzburg, Würzburg, Deutschland; 3 Institut für Evidenz in der Medizin, Universitätsklinikum Freiburg & Medizinische Fakultät, Universität Freiburg, Freiburg, Deutschland; 4 Cochrane Deutschland, Cochrane Deutschland Stiftung, Freiburg, Deutschland; 5 Abteilung für Medizinische Psychologie und Medizinische Soziologie, Universitätsklinikum Leipzig AöR, Leipzig, Deutschland; 6 Fakultät für Medizin, Klinikum rechts der Isar, Abteilung für Nephrologie, Technische Universität München, München, Deutschland; 7 Fakultät für Medizin, TUM Medical Education Center, Technische Universität München, München, Deutschland; 8 Klinik für Anästhesiologie, Universitätsmedizin Göttingen, Göttingen, Deutschland; 9 http://www.covid-evidenz.de

**Keywords:** Implementierung, Evidenzbasierte Medizin, COVID-19, Pandemie, Implementation, Evidence based medicine, COVID-19, Pandemic

## Abstract

**Hintergrund:**

Das COVID-19-Evidenz-Ökosystem (CEOsys) identifiziert, bewertet und fasst Ergebnisse wissenschaftlicher Studien in Evidenzsynthesen im Kontext von COVID-19 zusammen. Diese Evidenzsynthesen werden genutzt, um konkrete Handlungsempfehlungen abzuleiten und Leitlinien zu erstellen.

**Zielsetzung:**

Vorbereitung der am besten geeigneten Verteilung von Evidenzsynthesen im Rahmen der Aufgaben des CEOsys-Projekts.

**Methode:**

Für Deutschland wurde eine Befragung hinsichtlich des intensivmedizinischen Personals priorisierter Themenbereiche, Wünschen zu Layout, Plattform der Bekanntmachung von Evidenzsynthesen und Vertrauenswürdigkeit von Institutionen mit kategorialen Antwortmöglichkeiten durchgeführt. Die Umfrage erfolgte online und wurde per E‑Mail lokal und über die DIVI verteilt.

**Ergebnisse:**

Von 317 Befragten, die die Umfrage starteten, vervollständigten 200 den Fragebogen. Knappe Zeit und fehlender Zugriff, unzureichende Erfahrung bzw. Unsicherheit im Umgang mit Evidenzsynthesen wurden als Barriere für Wissenserwerb benannt. Das aktive Herantragen von Informationen wird bevorzugt („Push-Strategie“). Als Format werden Kurzversion, Übersichten mit Algorithmen und Webinare prioritär gewünscht. Webseiten öffentlicher Einrichtungen, Fachjournalartikel und E‑Mail-Newsletter sollen auf neue Evidenz aufmerksam machen. Fachgesellschaften und dem Robert Koch Institut werden in der Pandemie mehrheitlich Vertrauen geschenkt. Priorisierte Themen der Befragten sind Langzeitfolgen der Erkrankung, Schutz des medizinischen Personals und Invasivität der Beatmungstherapie.

**Schlussfolgerung:**

Evidenzsynthesen sollten aktiv an Zielgruppen herangetragen werden. Inhalte sollten übersichtlich, kurz (Algorithmen, Kurzversion, Webinare) und frei verfügbar sein. Webseiten, E‑Mail-Newsletter und medizinische Journale, aber auch Fachgesellschaften und das Robert Koch-Institut sollten auf Evidenzsynthesen hinweisen.

**Zusatzmaterial online:**

Zusätzliche Informationen sind in der Online-Version dieses Artikels (10.1007/s00101-021-01037-z) enthalten.

## Abstract (en)

**Background:**

In the context of COVID-19, the German CEOsys project (COVID-19 Evidenz Ökosystem, www.covid-evidenz.de) identifies, evaluates and summarizes the results of scientific studies to obtain evidence on this disease. The evidence syntheses are used to derive specific recommendations for clinical practice and to contribute to national guidelines. Besides the necessity of conducting good quality evidence syntheses during a pandemic, just as important is that the dissemination of evidence needs to be quick and efficient, especially in a health crisis. The CEOsys project has set itself this challenge.

**Objective:**

Preparing the most suitable distribution of evidence syntheses as part of the CEOsys project tasks.

**Methods:**

Intensive care unit (ICU) personnel in Germany were surveyed via categorical and free text questions. The survey focused on the following topics: evidence syntheses, channels and strategies of distribution, possibility of feedback, structure and barriers of dissemination and trustworthiness of various organizations. Profession, qualification, setting and size of the facility were recorded. Questionnaires were pretested throughout the queried professions (physician, nurse, others). The survey was anonymously carried out online through SosciSurvey® and an e‑mail was sent directly to 940 addresses. The survey was launched on 3 December, a reminder was sent after 14 days and it ended on 31 December. The survey was also announced via e‑mail through DIVI.

**Results:**

Of 317 respondents 200 completed the questionnaire. All information was analyzed including the responses from incomplete questionnaires. The most stated barriers were lack of time and access. Especially residents and nurses without specialization in intensive care mentioned uncertainty or insufficient experience in dealing with evidence syntheses as a barrier. Active distribution of evidence syntheses was clearly preferred. More than half of the participants chose websites of public institutions, medical journals, professional societies and e‑mail newsletters for drawing attention to new evidence syntheses. Short versions, algorithms and webinars were the most preferred strategies for dissemination. Trust in organizations supplying information on the COVID-19 pandemic was given to professional societies and the Robert Koch Institute (RKI) as the German governmental institute for infections and public health. The respondents‘ prioritized topics are long-term consequences of the disease, protection of medical personnel against infection and possibilities of ventilation treatment.

**Conclusion:**

Even though universally valid, evidence syntheses should be actively brought to the target audience, especially during a health crisis such as the COVID-19 pandemic with its exceptional challenges including lack of time and uncertainties in patient care. The contents should be clear, short (short versions, algorithms) and with free access. E‑mail newsletters, websites or medical journals should continuously report on new evidence syntheses. Professional societies and the governmental institute for infections and public health should be involved in dissemination due to their obvious trustworthiness.

## Hinführung

Wissenschaftliche Erkenntnisse zu COVID-19 aus Primärstudien mehren sich täglich. Deren systematische Bewertung und die Erstellung von Evidenzsynthesen erscheinen insbesondere vor dem Hintergrund der anhaltenden Pandemie vordringlich, um schnellstmöglich sinnvolle, evidenzbasierte Therapiekonzepte zu etablieren.

## Hintergrund und Fragestellung

Aus diesem Grund wurde im September 2020 das Verbundprojekt COVID-19-Evidenz-Ökosystem (CEOsys) zur Verbesserung von Wissensmanagement und -translation im Rahmen des Nationalen Forschungsnetzwerks der Universitätsmedizin (NUM) initiiert (www.covid-evidenz.de) und vom Bundesministerium für Bildung und Forschung finanziell gefördert.

Die alleinige Erstellung von Evidenzsynthesen und die Formulierung von Handlungsempfehlungen im Rahmen von Leitlinien führt bekanntermaßen jedoch nicht zu einer veränderten bzw. gebesserten klinischen Versorgung [1]. Hingegen müssen spezifische Implementierungsstrategien entwickelt werden, welche durch eine entsprechende Analyse und Adressierung von Barrieren für die jeweiligen Zielgruppen angepasst werden [2]. Die Aufbereitung und Verbreitung von Wissen stellen einen wichtigen Teilaspekt der Implementierung dar und soll hiermit untersucht werden.

Hierzu führten wir im Dezember 2020 eine onlinebasierte Umfrage zu Barrieren und Präferenzen für die Disseminierung von Evidenzsynthesen unter intensivmedizinischem Personal, das an COVID-19 erkrankte Patienten betreut, durch.

## Studiendesign und Untersuchungsmethoden

Die Erstellung des Fragebogens erfolgte im Rahmen von CEOsys an mehreren Universitätskliniken Deutschlands (federführend Würzburg, TU München und Leipzig). Um die schnelle Erhebung und Auswertung in der Pandemiesituation zu ermöglichen, wurde eine Online-Umfrage mit vorwiegend kategorialen Antwortmöglichkeiten erstellt. In diesem Kontext erschienen die Fragestellungen hinsichtlich priorisierter Themenbereiche, Kommunikationskanäle, Maßnahmen und Strategien zu Informations- und Evidenzvermittlung sinnvoll. Weiterhin wurde die gewünschte Struktur von Kurzzusammenfassungen, Implementierungsbarrieren und Inanspruchnahme einer Feedback-Option an die Verfasser von Evidenzsynthesen erfasst. Je nach Frage konnten eine bis 5 Optionen gewählt werden. Bei der Mehrzahl der Fragen war es möglich, über ein Freitextfeld in der Alternativantwort „Sonstiges“ die eigene Antwort genauer auszuführen. Die Freitextantworten konnten überwiegend bestehenden Kategorien zugeordnet werden. Wo dies nicht möglich war, wurden neue Kategorien gebildet. Außerdem sollten aus einer Auswahl von 7 vorgegebenen Institutionen mindestens 3 und maximal 5 Institutionen hinsichtlich ihrer Vertrauenswürdigkeit zur Dissemination in der Pandemiesituation gewählt werden. Berufsgruppe, Ausbildungsstand, Setting und Größe des Krankenhauses wurden erfasst, wohingegen soziodemografische Faktoren wie Alter und Geschlecht aus Datenschutzgründen nicht erhoben wurden. Adressaten waren das komplette Behandlungsteam auf Intensivstationen. Es wurde keine Mindestteilnehmerzahl oder spezifische Verteilung der Berufsgruppen festgelegt. Der vollständige Fragebogen ist im Supplement einsehbar.

Die Fragebogen wurden hinsichtlich Plausibilität und Verständlichkeit durch mehrere Vertreter*innen der adressierten Berufsgruppen (Ärzt*innen, Pfleger*innen und weiteres medizinisches Assistenzpersonal) getestet. Die Studie wurde unter Beachtung der Deklaration von Helsinki entworfen und der Ethikkommission der Universität Würzburg (Az: 2020-219/20) vorgelegt, die aufgrund der freiwilligen Teilnahme ohne Interventionscharakter keine weitere Prüfung verlangte. Die hinzugezogene Abteilung für Datenschutz erhob keinerlei Einwände (Korrespondenz vom 23.10.2020). Die Umfrage wurde mit SosciSurvey® (Fa. SosciSurvey GmbH, München, Deutschland) entworfen und durchgeführt.

Die Verbreitung des Umfrage-Links erfolgte über interne Kommunikationswege innerhalb von CEOsys an alle im Projekt beteiligten Intensivmediziner*innen. Außerdem wurden weitere lokale Kliniken mit Regel‑, Schwerpunkt- und Maximalversorgungsauftrag der Studieninitiator*innen über die Durchführung informiert und um Teilnahme und Verbreitung des Links innerhalb der Abteilungen an alle Mitarbeiter*innen gebeten. Insgesamt wurde der Link an 940 E‑Mail-Adressen direkt versandt. Ergänzend wurde die Studie von der Deutschen Interdisziplinären Vereinigung für Intensivmedizin (DIVI) per E‑Mail beworben. Start der Umfrage war der 03.12.2020. Es erfolgte nach 2 Wochen eine Erinnerung per E‑Mail. Die Umfrage wurde, um die Disseminationsstrategie von CEOsys rasch festlegen zu können, zum 31.12.2020 geschlossen.

Die Auswertung der Daten erfolgte mit SPSS 26 (IBM) und Excel (Microsoft). Die Teilnehmerzahlen variieren nach betrachteter Frage und werden im Laufe des Fragebogens geringer. Die relativen Häufigkeiten beziehen sich auf die Teilnehmer*innen einer Berufsgruppe, die die jeweilige Frage bearbeitet haben. Bei der Auswertung wurde sich an der Checkliste zur Auswertung von Online-Umfragen orientiert [3].

## Ergebnisse

### Studienpopulation

Von 668 Teilnehmer*innen, die die Umfrage aufgerufen haben, hatten 317 Personen mindestens eine Frage vollständig ausgefüllt. Aufgrund der geringen Gruppengröße „nichtärztliches medizinisches Personal im Rettungsdienst“ (*n* = 2) und „anderes nichtärztliches medizinisches Fachpersonal (z. B. MTA)“ (*n* = 12) wurden diese nicht in die Auswertung einbezogen. So ergeben sich für die Abfrage priorisierter Themenfelder eine Teilnehmerzahl von 303 Personen, im Umfrageteil zur Implementierung eine maximale Teilnehmerzahl von 236 Personen sowie in den Fragen zu Feedback und Informationsverteilung eine Teilnehmerzahl von 200 Personen.

Teilgenommen haben 186 Ärzt*innen, davon 69 Ärzt*innen in Weiterbildung und 117 Fachärzt*innen sowie 117 Pflegekräfte, davon 39 Gesundheits- und Krankenpfleger*innen sowie 78 Pflegekräfte mit Fachweiterbildung für Intensivmedizin. Den Fragebogen abgeschlossen haben 130 Ärzt*innen (davon 82 Fachärzt*innen) und 70 Pflegekräfte (davon 46 Pflegekräfte mit Fachweiterbildung für Intensivmedizin). Die Mehrzahl der Teilnehmer*innen war an einem Krankenhaus der Maximalversorgung (*n* = 238) tätig. Personal aus Kliniken der Regelversorgung (*n* = 39), Schwerpunktversorgung (*n* = 32), Fachkrankenhaus (*n* = 4) und sonstigen Einrichtungen (*n* = 4) nahm in deutlich geringerem Anteil an der Umfrage teil. Der Großteil der Befragten (*n* = 214, 70,6 %) hatte keine leitende Funktion inne.

Hinsichtlich des Krankenhausversorgungsgrades und leitender Funktion ergaben sich in den aufgeführten Fragen keine relevanten Unterschiede. Das Antwortverhalten der Berufsgruppen unterschied sich teilweise erheblich, auf das im Folgenden eingegangen werden soll.

### Barrieren für den Wissenserwerb

**Figure 1:**
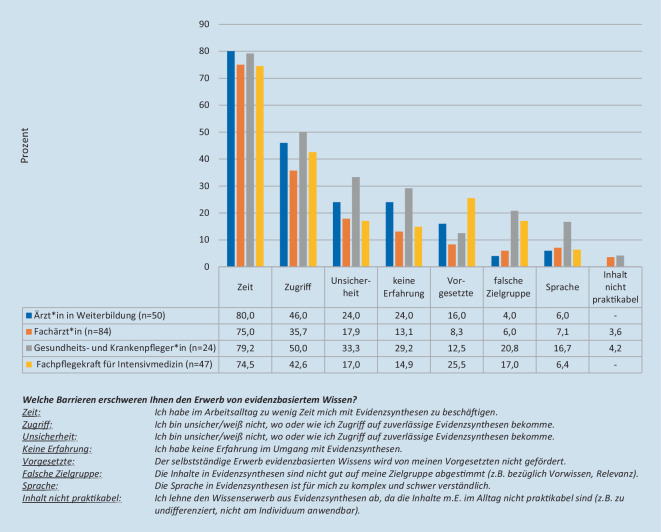

Die am häufigsten genannten Barrieren sind fehlende Zeit sowie kostenpflichtiger und umständlicher Zugang zu Evidenzsynthesen. Nachfolgend wurden Unsicherheit in der Bewertung von Evidenzsynthesen und fehlende Erfahrung im Umgang mit Evidenzsynthesen angegeben. Dies kommt insbesondere in der Gruppe der Assistenzärzt*innen und Pflegenden ohne Fachweiterbildung Intensivpflege zum Ausdruck. Bemerkenswert ist auch, dass sich Pflegende auf der Intensivstation nahezu 4‑mal häufiger nicht als Zielgruppe durch bisherige Evidenzsynthesen angesprochen fühlen (Abb. 1).

### Disseminierungsstrategie

Zur Informationsvermittlung zieht mit 74,6 % des ärztlichen Personals (*n* = 130) und 68,6 % des pflegerischen Personals (*n* = 70) die deutliche Mehrheit eine Push-Strategie, also das aktive Herantragen und Vermitteln der Evidenzsynthesen, gegenüber einer Pull-Strategie (Abrufen von Informationen durch den Nutzer) vor.

**Figure 2:**
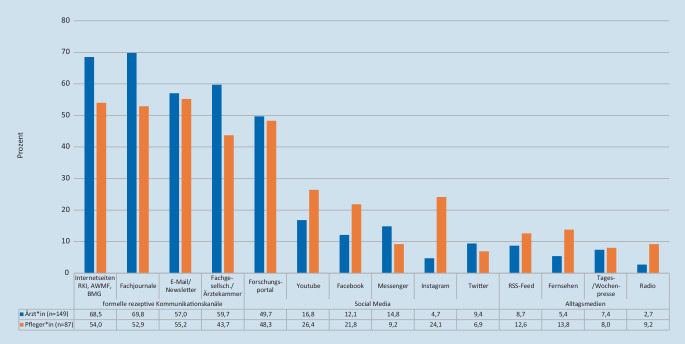

An Kanälen zum Wissenserwerb wünschen sich alle befragten ärztlichen und pflegerischen Kolleg*innen (*n* = 236) nahezu gleichermaßen Fachjournale (63,6 %), Fachgesellschaften (53,8 %), Internetseiten öffentlicher Stellen (63 %) (wie z. B. Robert Koch-Institut oder Bundesministerium für Gesundheit) oder E‑Mail-Newsletter (56,4 %). Bei den Social-Media-Kanälen werden insbesondere Forschungsportale (z. B. *ResearchGate®* 49,2 %) häufig genannt. Social-Media-Kanäle wie *YouTube* oder *Instagram* werden eher selten gewählt, wobei diese von Pflegekräften häufiger präferiert werden. Klassische Medien (Radio, Fernsehen, Tages- oder Wochenpresse) spielen in der Gruppe der Befragten eine untergeordnete Rolle (Abb. 2).

In der Freitextoption wurden in geringer Quantität interne Kommunikationskanäle, digitale Systeme zur Unterstützung in klinischen Entscheidungen und Information über „Free Open Access Meducation – FOAM“ genannt. Vereinzelt wurden Blogs, persönliche berufliche Kontakte, Fortbildungsplattformen und bibliografische Datenbanken als weitere Kommunikationskanäle gewünscht.

Bei den Strategien zum Wissenserwerb spielen insbesondere Kurzfassungen, Handlungsalgorithmen, Webinare und Checklisten eine vorrangige Rolle. Nachfolgend finden sich Artikel in Fachjournalen, Apps und die Langfassung mit deutlichem Unterschied häufiger bei den Ärzt*innen. Hingegen werden von den Pflegenden vorrangig Videos (44,4 % aller Pflegekräfte) sowie ferner Weiterbildungen am Arbeitsplatz und Podcasts häufiger gewünscht (Tab. 1).

**Table 1:** 

		Ärzt*in in Weiterbildung (*n* = 51)	Fachärzt*in (*n* = 93)	Gesundheits- und Krankenpfleger*in (*n* = 29)	Fachpflegekraft für Intensivmedizin (*n* = 53)
		*n* (%)	*n* (%)	*n* (%)	*n* (%)
Online- oder Print-Material zum aktiven Wissenserwerb (z. B. CEOsys-Website. spezielle Apps, Journale)	Übersichten & Handlungsalgorithmen	36 (70,6)	60 (64,5)	8 (27,6)	30 (56,6)
	Kurzfassung	20 (39,2)	56 (60,2)	13 (44,8)	31 (58,5)
	Fachjournale	13 (25,5)	44 (47,3)	5 (17,2)	14 (26,4)
	App	20 (39,2)	33 (35,5)	8 (27,6)	8 (15,1)
	Langfassung	11 (21,6)	33 (35,5)	5 (17,2)	6 (11,3)
	Fortbildungsvortrag	7 (13,7)	16 (17,2)	6 (20,7)	14 (26,4)
	Poster	1 (2,0)	5 (5,4)	4 (13,8)	10 (18,9)
Onlinematerial für passiven Wissenserwerb (jederzeit abrufbar)	Podcast	15 (29,4)	32 (34,4)	10 (34,5)	13 (24,5)
	Videos	17 (33,3)	15 (16,1)	10 (34,5)	22 (41,5)
	Vertonte PPP	12 (23,5)	21 (22,6)	7 (24,1)	16 (30,2)
Weiterbildung	Webinar	25 (49,0)	40 (43,0)	10 (34,5)	25 (47,2)
	Weiterbildung am Arbeitsplatz	14 (27,5)	10 (10,8)	11 (37,9)	12 (22,6)
	Weiterbildung (Präsenz)	6 (11,8)	5 (5,4)	7 (24,1)	8 (15,1)
	Kongresse (Präsenz)	3 (5,9)	11 (11,8)	3 (10,3)	7 (13,2)
	Digitaler Journalclub	1 (2,0)	8 (8,6)	1 (3,4)	–
Dokumentation und Qualitätsmanagement	Checklisten	18 (35,3)	23 (24,7)	12 (41,4)	19 (35,8)
	Dokumentationssystem	5 (9,8)	10 (10,8)	4 (13,8)	7 (13,2)
	Audit, hausinterne Expertise	11 (21,6)	5 (5,4)	–	8 (15,1)
	Vergleich mit anderen Einrichtungen	–	7 (7,5)	1 (3,4)	4 (7,5)
	Audit, externe Expertise	1 (2,0)	1 (1,1)	–	2 (3,8)
	Belohnungssystem	1 (2,0)	1 (1,1)	1 (3,4)	1 (1,9)

Hinsichtlich der Struktur von Zusammenfassungen (Grafik, Stichpunkte, Fließtext) ließ sich keine eindeutige Präferenz der Befragten erkennen, während bezüglich der Aufbereitung der Großteil der Teilnehmer*innen (74 %, *n* = 204) eine kombinierte digitale und analoge Präsentation bevorzugte. Eine rein digitale Lösung halten nur 22 % der Befragten für ausreichend. Eine Möglichkeit zum Feedback direkt an die Ersteller von Evidenzsynthesen würde von 8,5 % der Befragten (*n* = 200) niemals genutzt.

### Vertrauenswürdigkeit bestimmter Institutionen und gewünschte Themen der COVID-19-Pandemie

**Figure 3:**
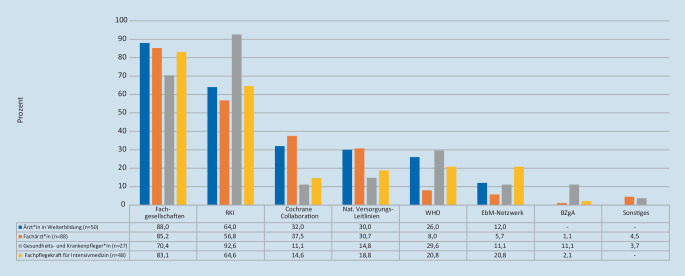

Fachgesellschaften und das Robert Koch-Institut werden von der deutlichen Mehrheit der Befragten als vertrauenswürdig eingeschätzt, bei Ärzt*innen gefolgt von der Cochrane Collaboration. Die Pflegenden wählten Letztere deutlich seltener aus (Abb. 3).

**Figure 4:**
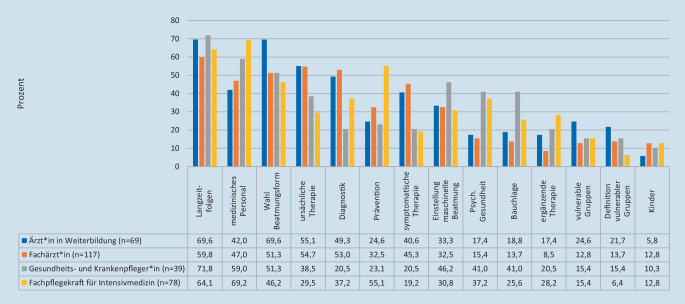

Das Interesse der Befragten zu Themen, die von Evidenzsynthesen behandelt werden sollten, gilt vorrangig den Langzeitfolgen, dem Schutz des medizinischen Personals und der Wahl der Beatmungsform. Diagnostik, kausale und symptomatische Therapie werden häufiger bei Ärzt*innen genannt, während sich Pflegekräfte besonders für die psychische Gesundheit interessieren. Die Bauchlagerungstherapie hat bei 41 % der Pflegekräfte ohne Fachweiterbildung im Gegensatz zu den anderen Berufsgruppen einen sehr hohen Stellenwert (Abb. 4).

## Diskussion

Unsere Umfrage zu Disseminierungspräferenzen und -barrieren unter intensivmedizinischem Personal ergab, dass Evidenzsynthesen aktiv an Zielgruppen herangetragen werden sollten. Darüber hinaus sollten Inhalte übersichtlich, kurz (Algorithmen, Kurzversion, Webinare) und frei verfügbar sein. Webseiten, E‑Mail-Newsletter und medizinische Journale, aber auch Fachgesellschaften und das Robert Koch-Institut sollten, weil als besonders vertrauenswürdige Institutionen eingestuft, auf Evidenzsynthesen zu COVID-19 hinweisen.

Die Ergebnisse unserer Umfrage legen nahe, dass vorrangig der empfundene Zeitmangel und ein beschränkter bzw. kostenpflichtiger Zugriff sowie die fehlende Erfahrung bzw. Unsicherheit im Umgang mit Evidenzsynthesen die hauptsächliche Barrieren bei der Vermittlung bzw. dem Benutzen von Evidenzsynthesen im intensivmedizinischen Behandlungsteam darstellen. Dies deckt sich mit aktuellen, bestehenden Übersichtsarbeiten zu Barrieren von Leitlinienimplementierung [2] und könnte sich im Rahmen der Pandemie bei Personalmangel und einem hohen Aufkommen an kritisch-kranken Patienten noch verschärfen.

Der Großteil der Befragten wünscht die aktive Vermittlung von Inhalten, wie dies in der Literatur auch vor der derzeitigen Pandemiesituationen vorbeschrieben ist [4, 5]. Kommunikationskanäle über Webseiten staatlicher Institute (Bundesministerium für Gesundheit, Robert Koch-Institut), Fachjournale, Fachgesellschaften, E‑Mail-Newsletter oder akademische Social-Media-Plattformen werden prioritär von allen Befragten genannt. Diese Kanäle stellen entsprechend [6] den der jeweiligen Zielgruppe angepassten Verbreitungsweg dar und könnten somit für eine schnellere Dissemination neuen Wissens in einer Pandemiesituation helfen. Im Unterschied zeigt sich jedoch bei den Pflegekräften die relative Präferenz von klassischen Social-Media-Kanälen (vorrangig *YouTube, Instagram* und *Facebook*) als weitere, präferierte Informationskanäle. Für diese Zielgruppe erscheint somit die Produktion hochwertiger Inhalte auf diesen Plattformen relevant, nicht zuletzt, um den bereits existierenden Fehlinformationen eine fundierte Wissensbasis entgegensetzen zu können [7].

Die Strategie zum Wissenserwerb sollte anhand unserer Ergebnisse berufsgruppenspezifisch erfolgen. Ärzt*innen sollten Informationen mittels Algorithmen, Kurzfassungen oder auch über Artikel in Fachjournalen erhalten. Die ausführliche Langfassung spielt für Fachärzte nach wie vor eine bedeutende Rolle. Online verfügbare Formate wie Webinare oder Podcasts werden gegenüber Präsenzveranstaltungen favorisiert.

Pflegekräfte wünschen sich ebenfalls eine Kurzversion und Handlungsalgorithmen. Neben Webinaren kann hier auch die Produktion von Videos und Podcasts in der Wissensvermittlung besonders hilfreich sein. Bemerkenswert ist der deutliche Wunsch nach Weiterbildung direkt am Arbeitsplatz. Eine maßgeschneidertes Implementierungskonzept [8] gilt bekanntermaßen als vorteilhaft und sollte im weiteren Verlauf evaluiert werden. Insbesondere Webinare als interaktive Plattform zur Vermittlung komplexer Inhalte und als Alternative zu Präsenzveranstaltungen aufgrund der Kontaktbeschränkungen stoßen bei der Mehrheit der Befragten auf Akzeptanz und wurden zuvor schon als effektives Mittel der Evidenzvermittlung in der Pandemie beschrieben [9]. Vor der Pandemie konnten diese Werkzeuge nicht als effizient bewertet werden [10]. Eventuell könnte sich dies durch den Einfluss der Digitalisierung in der Pandemie geändert haben und sollte weiter untersucht werden.

Fachgesellschaften und das Robert Koch-Institut genießen insbesondere im Kontext der COVID-19-Pandemie über alle Berufsgruppen hinweg eine breite Vertrauensbasis, sodass diese für diesen Implementierungsprozess eine wichtige Rolle spielen und idealerweise bei der Dissemination von Evidenzsynthesen eingebunden werden sollten [6]. Diese Ergebnisse sind zeitlich und inhaltlich im Kontext der aktuellen Pandemie zu sehen. Eine solche Wichtung öffentlicher Einrichtung für die Implementierung klinischer Leitlinien war vor der Pandemie in der Literatur nicht vorbeschrieben. Hier sollten jedoch die aufwendig aufgearbeiteten Evidenzsynthesen mit der gebührenden Wichtung in den Gesamtkontext eingebettet werden und nicht gleichwertig zu methodisch minderwertigen Sachverhalten präsentiert werden.

Zu den am häufigsten interessierenden Themenkomplexen zählen COVID-19-Langzeitfolgen, Eigenschutzmaßnahmen und Wahl der Beatmungsform. Dies scheint die zu Beginn der Pandemie kontroverse Diskussion zur Gefahr der nichtinvasiven Beatmung aufgrund von Aerosolbildung widerzuspiegeln [11]. Ärzt*innen haben großes Interesse an symptomatischer oder kausaler Therapie, wohingegen sich Pflegekräfte häufig für psychische Langzeitfolgen interessieren. Pflegekräfte zeigen für Themen der Lagerungstherapie oder der Respiratoreinstellung ein größeres Interesse als die Ärzt*innen. Diese Themen sollten für kommende Evidenzsynthesen durch CEOsys bearbeitet werden, um den Informationsbedarf zu decken.

Bei der hier vorliegenden Umfrage ist von einer willkürlichen Stichprobe des intensivmedizinischen Behandlungsteams auszugehen. Hier zeigt sich deutlich, dass der übermäßige Anteil aus der Gruppe der Ärzt*innen aus Maximalversorgern resp. Universitätsklinika stammte. Jedoch konnten auch Pflegekräfte in ausreichender Anzahl gewonnen werden. Beachtenswert ist, dass der Anteil des fachlich spezialisierten Personals höher ist (Ärzt*innen und Pflegekräfte) und dies zumindest im Fall der Pflege nicht die übliche Besetzung einer Intensivstation widerspiegelt [12]. Die Beteiligung an der freiwilligen Umfrage fiel auch für eine Online-Umfrage eher gering aus [13], wobei letztlich nicht sicher festgestellt werden kann, wie viele Personen der Umfrage-Link erreichte. Aufgrund der geringen Beteiligung und insbesondere der Zusammensetzung der Teilnehmer ist von einer nichtrepräsentativen Umfrage auszugehen. Ursachen für die geringe Teilnahme könnte fehlende Zeit aufgrund der parallel hohen Arbeitsbelastung durch die zweite Welle der COVID-19-Pandemie in Deutschland im Dezember 2020 sein. Auch die überwiegende Beteiligung von Ärzt*innen und Mitarbeiter*innen aus Maximalversorgern könnte an der vorhergehenden Kenntnis des Projekts bzw. Bereitschaft zur Mitwirkung verankert sein und an der primären Verteilung über Ärzt*innen an Universitätskliniken selbst liegen. Letztlich war das Ziel der Umfrage, zügig eine Orientierung für die Disseminationsstrategie für CEOsys zu erhalten, um eine Relevanz für die Bewältigung der Pandemie darzustellen. Zugunsten einer schnellen Umsetzung wurde sich deshalb bewusst für eine zeitlich begrenzte Online-Umfrage entschieden. Nichtärztliches und -pflegerisches Personal beteiligte sich nur in geringem Ausmaß an der Umfrage, sodass zu dieser Zielgruppe keine sicheren Aussagen getroffen werden können. Das Format der kategorialen Antwortmöglichkeiten schränkt die erreichbare Detailliertheit der Aussagen ein, es war jedoch jederzeit die Eingabe einer offenen Antwort möglich und wurde auch entsprechend genutzt.

## Fazit für die Praxis

Ableitungen aus unserer nichtrepräsentativen Umfrage:

- Evidenzsynthesen im Kontext der COVID-19-Pandemie sollten frei verfügbar, leicht zugänglich und inhaltlich schnell erfassbar sein.
- Der unerfahrene Nutzer sollte eine Hilfestellung zum Verständnis vom Zweck und zur Methode von Evidenzsynthesen erhalten.
- Die Information soll aktiv an die Zielgruppe herangetragen werden.
- Die Vermittlung der Inhalte sollte berufsgruppenspezifisch erfolgen. Ärzt*innen bevorzugen Algorithmen und eine Kurzfassung, wohingegen Pflegekräfte neben einer Kurzfassung und Checklisten auch Videos und Podcasts favorisieren. Webinare sind in beiden Gruppen ähnlich beliebt.
- Bei Texten sollte eine Möglichkeit zum Ausdrucken gegeben sein.
- Für die Verteilung neuer Evidenz sind neben E-Mail-Newslettern auch intensivmedizinische Fachgesellschaften und das Robert-Koch-Institut zu empfehlen.
- Für den deutschen Versorgungsraum entwickelt CEOsys auf Grundlage der Ergebnisse erstmals eine solche adaptierte Disseminierungsstrategie.

## Supplementary Information




