# Supplementary material for: COVID-19 pandemic: preferences and barriers for dissemination of evidence syntheses: Survey of intensive care personnel in Germany
Source: Anaesthesist. 2021 Sep 21;71(4):281–90. [Article in German] doi: 10.1007/s00101-021-01037-z (PMC8454015; doi:10.1007/s00101-021-01037-z)
Supplement: Supplementary file 1 [file 101_2021_1037_MOESM1_ESM.pdf]

Zusatzmaterial zum Beitrag

„COVID-19 Pandemie: Präferenzen und Barrieren für die Disseminierung von Evidenzsynthesen. Befragung des intensivmedizinischen Personals in Deutschland“ von Seeber C, Popp M, Meerpohl JJ et al. (2021) in Der Anaesthesist.

Beitrag und Zusatzmaterial stehen Ihnen auf www.springermedizin.de zur Verfügung. Bitte geben Sie dort den Beitragstitel in die Suche ein.

Fragebogen

Nr.	Frage	**Bedingungen**
1	Welcher Berufsgruppe sind Sie zugehörig?	
	1) Ärzt*in in Weiterbildung<br>2) Fachärzt*in<br>3) Gesundheits- und Krankenpfleger*in<br>4) Fachpflegekraft für Intensivmedizin<br>5) Nicht-ärztliches medizinisches Personal im Rettungsdienst<br>6) Anderes nicht-ärztliches medizinisches Fachersonal (z.B. MTA)	*Muss mit 1 Option beantwortet werden*
2	Haben Sie eine organisatorische oder leitende Position inne?	
	1) Ja<br>2) nein	*Muss mit 1 Option beantwortet werden*
3	In welcher Art der Einrichtung sind Sie tätig?	
	1) Krankenhaus der Grund-/Regelversorgung<br>2) Krankenhaus der Maximalversorgung<br>3) Krankenhaus der Schwerpunktversorgung<br>4) Fachkrankenhaus<br>5) Sonstiges → [*Freitext*]	*Muss mit 1 Option beantwortet werden;*<br><br>*falls Freitext-Option gewählt wird, muss konkrete Angabe als Freitext eingegeben werden*
4	Bitte priorisieren Sie in welchen Pandemie-bezogenen Themenbereichen Sie sich besonders Informationen wünschen.	
	1) medizinisches Personal (Ärzteschaft, Pflegekräfte, andere Gesundheits-fachberufe)<br>2) Kinder<br>3) vulnerable Gruppe<br>4) Definition vulnerabler Gruppe<br>5) Prävention (Isolierungsmaßnahmen, Schutzkleidung)<br>6) Diagnostik (Testkriterien, intensiv-medizinische Diagnostik bei COVID-19)<br>7) Langzeitfolgen von COVID-19<br>8) Psychische Gesundheit während COVID-19<br>9) medikamentöse (ursächliche) Therapie<br>10) medikamentöse (symptomatische) Therapie	*Mind. 1 Option, maximal 5 anwählen*

	11) ergänzende Therapiemaßnahmen (z.B. Mobilisierung, Physio- oder Ernährungstherapie)<br>12) Entscheidungskriterien zur Wahl der Beatmungsform (NIV, Intubation, ECMO)<br>13) Einstellungen/Variationen bei maschineller Beatmung (z.B. PEEP,<br>NIV Helm vs. Maske)<br>14) Anwendung der Bauchlage bei Intensivpatient*innen (mit und ohne invasive Beatmung)<br>15) Vermeidung von Nebenwirkungen bei medikamentöser Behandlung	
5	Welche weiteren Fragestellungen bezüglich COVID-19 in der Intensivmedizin, neben den genannten Themengebieten, sind hinsichtlich Ihrer täglichen Arbeit besonders relevant und sollten in einem umfassenden Wissens- und Evidenzökosystem behandelt werden?	
	[*Freitext*]	*optional*
6	Welche der folgenden Plattformen und Kanäle nutzen Sie oder würden Sie nutzen, um auf neu generierte Evidenz/Behandlungsempfehlungen aufmerksam aufmerksam zu werden?	
	1) Twitter<br>2) Facebook<br>3) Instagram<br>4) Youtube<br>5) Messenger-Channel (z.B. Telegram)<br>6) Vernetzung in einem Forschungsportal (z.B. ResearchGate)<br>7) Sonstiges (Online/Social Media) → [*Freitext*]<br>8) automatisierter Newsfeed (RSS-Feed)<br>9) Email/Newsletter<br>10) Fachjournale<br>11) Fernsehen<br>12) Radio<br>13) Tages-/Wochenpresse<br>14) Internetseiten des Robert-Koch-Instituts, AWMF, Bundesgesundheits-ministerium u.ä.<br>15) Fachgesellschaften/Ärztekammer<br>16) Sonstiges (Kampagnen/Anzeigen) → [*Freitext*]	*Mind. 1 Option, maximal 5 anwählen;*<br><br>*falls Freitext-Option (7;16) gewählt wird, muss auch eine konkrete Angabe als Freitext eingegeben werden*
7	Gerade mit Blick auf die sich dynamisch entwickelnde Evidenz- und Wissenslage während einer Pandemie, welche Strategien sind <u>für Sie</u> am effektivsten, um Kenntnisse über konkrete Inhalte zu erlangen und praktisch umzusetzen?	
	1) Frei verfügbare Langfassung (Originalarbeit)<br>2) Frei verfügbare Kurzfassung (inhaltlich/sprachlich angepasst an Zielgruppe)<br>3) Übersichten mit Handlungsalgorithmen<br>4) Poster<br>5) Bereitstellen eines frei verwendbaren Fortbildungsvortrages<br>6) Beiträge in Fachjournalen<br>7) Mobile Unterstützungssystem für klinische Entscheidungen (z.B. Amboss-App, UpToDate-App)<br>8) Vertonte Power-Point-Präsentationen<br>9) Podcast in Form von Interviews mit Expert*innen oder verbaler Darstellung von Inhalten	*Mind. 1 Option, maximal 5 anwählen;*<br><br>*→ falls Freitext-Option (22) gewählt wird, muss auch eine konkrete Angabe als Freitext eingegeben werden*<br><br>*→ Filterfrage:*<br>*Nur wenn eine Option von 1, 2,3,4,6 oder 22*

	10) Videos in Form von Reportagen und audiovisueller Darstellung von Inhalten<br>11) Kongresse (Präsenzveranstaltung)<br>12) Fachspezifische Weiterbildungsveranstaltungen (Präsenzveranstaltung)<br>13) Weiterbildungsveranstaltungen direkt am Arbeitsplatz (Präsenzveranstaltung)<br>14) Webinar (Onlineformat mit Interaktionsmöglichkeit, findet zu einem definierten Zeitpunkt statt)<br>15) Digitaler Journalclub (Diskussion mit Expert*innen aus CEOsys mit der Möglichkeit Fragen zu stellen)<br>16) Erinnerungen und Qualitätsmarkern eingebunden in (digitales) Dokumentationssystem<br>17) Checklisten (z.B. am Patientenbett)<br>18) Audit und Feedback der täglichen Praxis durch hausinterne Expertise<br>19) Audit und Feedback der täglichen Praxis durch externe Expertise<br>20) individuelles Belohnungssystem<br>21) Qualitätsindikatoren veröffentlichen, um einen Vergleich mit anderen Einrichtungen zu ermöglichen<br>22) Sonstiges → [*Freitext*]	*gewählt wird, erscheint Frage 8*
8	Unabhängig von dem genauen Textformat, welche Art der Verbreitung von Lesematerial/Informationstexten sagt Ihnen am ehesten zu?	
	1) Artikel zum direkten Lesen rein digital auf einer Website/App zur Verfügung stellt.<br>2) Artikel als PDF mit Möglichkeit zum Ausdrucken auf einer Website zur Verfügung stellt.<br>3) analoges Printmaterial zur Verfügung stellt.	*Muss mit 1 Option beantwortet werden*
9	Müssten Sie sich für ein Format der Kurzzusammenfassung von Evidenzsynthesen entscheiden, welches wäre es?	
	Zusammenfassung mit Interpretation<br>1) Tabellarisch<br>2) Fließtext<br>3) Stichpunkte<br>4) Grafisch/Infografik<br>Zusammenfassung ohne Interpretation<br>5) tabellarisch<br>6) Fließtext<br>7) Stichpunkte<br>8) Grafisch/Infografik	*Muss mit mind. 1 Option beantwortet werden, maximal 3*
10	Welche Organisationen (Herkunft der Information) stufen Sie im Bezug auf Information zur medizinischen Therapie von COVID-19 als besonders vertrauenswürdig ein?	

	1) Cochrane Collaboration<br>2) Deutsches Netzwerk Evidenzbasierte Medizin (EbM-Netzwerk)<br>3) Fachgesellschaften (z.B. DIVI, DGAI, DGIIN)<br>4) Programm für Nationale Versorgungsleitlinien (Kooperation von Bundesärztekammer, KBV, AWMF)<br>5) World Health Organisation (WHO)<br>6) Robert-Koch-Institut<br>7) Bundeszentrale für gesundheitliche Aufklärung<br>8) Sonstiges → [*Freitext*]	*Muss mit mind. 1 Option beantwortet werden, maximal 3;*<br>*falls Freitext-Option (8) gewählt wird, muss auch eine konkrete Angabe als Freitext eingegeben werden*
11	Welche Strategie zur Informationsvermittlung, mit der das CEOsys Konsortium neu generierte Evidenzsynthesen und Leitlinien verbreiten wird, bevorzugen Sie?	
	1) Themen und Inhalte werden aktiv an Sie als Zielgruppe herangetragen;<br>z.B. per Email oder Social Media Meldung mit Link zum Volltext/Zusammenfassungen oder auch über Ihre Vorgesetzten bzw. organisatorische Leitung der Einrichtung, ohne dass Sie dafür aktiv werden müssen („Push Strategie“).<br>2) Themen und Inhalte sind frei verfügbar. Die Dauer, Frequenz und Detailtiefe der Informationsvermittlung hängt von Ihnen ab. Die Informationen werden anhand zuvor durch Ihr Feedback festgelegter Themen bereitgestellt („Pull Strategie“).	*Muss mit 1 Option beantwortet werden*
12	Werden Sie eine Feedback-Option, d.h. eine Möglichkeit zur Rückmeldung an das CEOsys Konsortium oder andere Organisationen, die Evidenzsynthesen/Leitlinien erstellen (z.B. AWMF-koordinierte bundesweite Leitlinienprojektgruppen), nutzen?	
	1) ja, werde ich<br>2) eher ja<br>3) eher nein<br>4) nein, werde ich nicht	*Muss mit 1 Option beantwortet werden*
13	Welche Barrieren erschweren Ihnen den Erwerb von evidenzbasiertem Wissen?	
	1) Ich habe im Arbeitsalltag zu wenig Zeit mich mit Evidenzsynthesen zu beschäftigen.<br>2) Der selbstständige Erwerb evidenzbasierten Wissens wird von meinen Vorgesetzten nicht gefördert.<br>3) Ich habe keine Erfahrung im Umgang mit Evidenzsynthesen.<br>4) Ich bin unsicher/weiß nicht, wo oder wie ich Zugriff auf zuverlässige Evidenzsynthesen bekomme.<br>5) Der Zugriff auf Evidenzsynthesen gestaltet sich zu umständlich (z.B. kostenpflichtige Abonnements/Mitgliedschaften).<br>6) Die Sprache in Evidenzsynthesen ist für mich zu komplex und schwer verständlich.<br>7) Die Inhalte in Evidenzsynthesen sind nicht gut auf meine Zielgruppe abgestimmt (z.B. bezüglich Vorwissen, Relevanz).<br>8) Ich lehne den Wissenserwerb aus Evidenzsynthesen ab, da die Inhalte m.E. im Alltag nicht praktikabel sind (z.B. zu undifferenziert, nicht am Individuum anwendbar).<br>9) Sonstiges → [*Freitext*]	*Muss mit mind. 1 Option beantwortet werden, maximal 3;*<br>*falls Freitext-Option (9) gewählt wird, muss auch eine konkrete Angabe als Freitext eingegeben werden*
